# Spatial social network research: a bibliometric analysis

**DOI:** 10.1007/s43762-022-00045-y

**Published:** 2022-07-09

**Authors:** Ling Wu, Qiong Peng, Michael Lemke, Tao Hu, Xi Gong

**Affiliations:** 1 Texas Research Data Center, Texas A&M University, College Station, USA; 2 Department of Computer Science, Northeastern University, Boston, USA; 3 Department of Social Sciences, University of Houston-Downtown, Houston, USA; 4 Department of Geography, Oklahoma State University, Stillwater, USA; 5 Department of Geography and Environmental Studies, University of New Mexico, Albuquerque, USA

**Keywords:** Social network, Social media, Geography

## Abstract

A restless and dynamic intellectual landscape has taken hold in the field of spatial social network studies, given the increasingly attention towards fine-scale human dynamics in this urbanizing and mobile world. The measuring parameters of such dramatic growth of the literature include scientific outputs, domain categories, major journals, countries, institutions, and frequently used keywords. The research in the field has been characterized by fast development of relevant scholarly articles and growing collaboration among and across institutions. The *Journal of Economic Geography*, *Annals of the Association of American Geographers*, and *Urban Studies* ranked first, second, and third, respectively, according to average citations. The United States, United Kingdom, and China were the countries that yielded the most published studies in the field. The number of international collaborative studies published in non-native English-speaking countries (such as France, Italy, and the Netherlands) were higher than native English-speaking countries. Wuhan University, the University of Oxford, and Harvard University were the universities that published the most in the field. “Twitter”, “big data”, “networks”, “spatial analysis”, and “social capital” have been the major keywords over the past 20 years. At the same time, the keywords such as “social media”, “Twitter”, “big data”, “geography”, “China”, “human mobility”, “machine learning”, “GIS”, “location-based social networks”, “clustering”, “data mining”, and “location-based services” have attracted increasing attention in that same time frame, indicating the future research trends.

## Introduction

Social science data typically consist of meanings, motives, definitions, and typification (Scott, 2000). In addition, the main types of social science data include attribute data and relational data (Scott, 1988). Attribute data involve the properties, qualities, or features which characterize individuals or groups, while relational data are the contacts, ties, and connections among individuals or groups. Network analysis is especially suitable to relational data, where the relations can be treated as the linkages among agents. Coming from textile metaphors, the term ‘network’ was integrated into the social science domains starting in the 1930s to indicate the interweaving relations how social actions, agents, and groups are organized. From the 1970s, the key and formal concepts of social network analysis emerged in social science domains and have triggered new modes of techniques capable of tackling the relational data. As another critical perspective indicating relations among social agents, geographical or spatial dimension was largely ignored until the 2000s. After that, more and more researchers delved into spatial social network analysis, and the corresponding literature base has been growing. Spatial social networks are typically treated as spatial transformations of social networks into maps (Tsou & Yang, 2016). In this context, the use of geospatial technologies in social network analysis has received growing attention. In particular, analyzing spatial social networks can reveal the spatial–temporal dynamics of information and link people’s online communications with real-world events (Yang et al., 2016; Ye & Liu, 2018).

As the use of spatial social network analysis has proliferated in the social sciences, its potential for explicating social phenomena has become increasingly realized, with implications for corresponding action strategies and policy solutions. For example, methodologies that integrate spatial and social network data have been applied in infectious disease and chronic disease epidemiology (Emch et al., 2012; Sun et al., 2018). In the domain of urban research in particular, these methodologies have been used for in the study of urban agglomerations, such as for transportation planning and air pollution mitigation (Song et al., 2018; Zhang et al., 2020; Zhu et al., 2021). However, as researchers increasingly consider spatial social network analysis, it is necessary to portray its research trends comprehensively (Ye & Andris, 2021). A nuanced understanding of these trends can serve several purposes, such as: (1) facilitating the sharing of research achievements in the field; (2) identifying emerging research directions; and (3) encouraging the continued development of research methods (Donthu et al., 2021). However, outside of a handful of studies that reflect on broader trends in this area, a general overview about spatial social network analysis does not exist. Thus, this paper addresses this persistent gap by conducting a review of network analysis from the perspective of spatial social science.

To portray research trends in the use of spatial social network analysis, a bibliometric analysis was conducted. Bibliometric analysis is a statistical approach to analyze relevant publications and understand the research trends in a particular domain (Garfield, 1970; Pritchard, 1969). In the current study, the purpose of this type of analysis is to identify the trends of publications and collaborations, as well as geographical and institutional distributions of scholarly outputs (Li et al., 2017). Furthermore, bibliometric network analysis, such as co-word analysis (Ding et al., 2001), co-citation analysis (He & Hui, 2002), co-authorship analysis (Glänzel & Schubert, 2004), and co-publication analysis (Schmoch & Schubert, 2008), was conducted in the current study to examine the relationships among authors, keywords, institutes, and countries.

This paper examines the research trends of publications on spatial social network analysis from the years 2000 to 2019. The aims of the study are to: (1) evaluate the research performance by country, institute, journal, subject category, and keyword; and (2) identify state-of-the art techniques and future research directions in spatial social network analysis.

## Methodology

### Data collection

The dataset was derived from the databases of the Science Citation Index Expanded (SCI-expanded) and Social Science Citation Index (SSCI) publications by the Web of Science covering the time period of 2000 to 2019. The following keywords, including TS (Topic) = (“social network*” OR “social media*”) AND (“spatial” OR “geography”), were employed to search all the archived documents for relevant publications. The selected publications include those keywords or close variants of those keywords (with *) in their titles, abstracts, or keywords. Information regarding titles, abstract, keywords, authors, institutions, and cited references was downloaded. The bibliographic search resulted in 2,721 publications. After deleting the records without complete authorship and publication year, 2,676 publications remain.

### Analysis tools

Bibliometric analysis was conducted to assess the trends of spatial social network analysis research in the scientific literature. In this study, we used the R package “Bibliometrix” and the VOSviewer (Aria & Cuccurullo, 2017; Van Eck & Waltman, 2009). The R package “Bibliometrix” provides an open-source package of bibliometrics and scientometrics. The VOSviewer is a free toolbox for developing bibliometric visualization and analyzing publication trends. Natural language processing methods are built into the VOSviewer package, which can be used to generate the term co-occurrence networks, network layouts, and network clusters. The VOSviewer software adopts a labelled circle to denote an element, where the circle size indicates the relative importance, and the same color represents the same cluster.

## Results and discussion

### Characteristics of publications

**Fig. 1:**
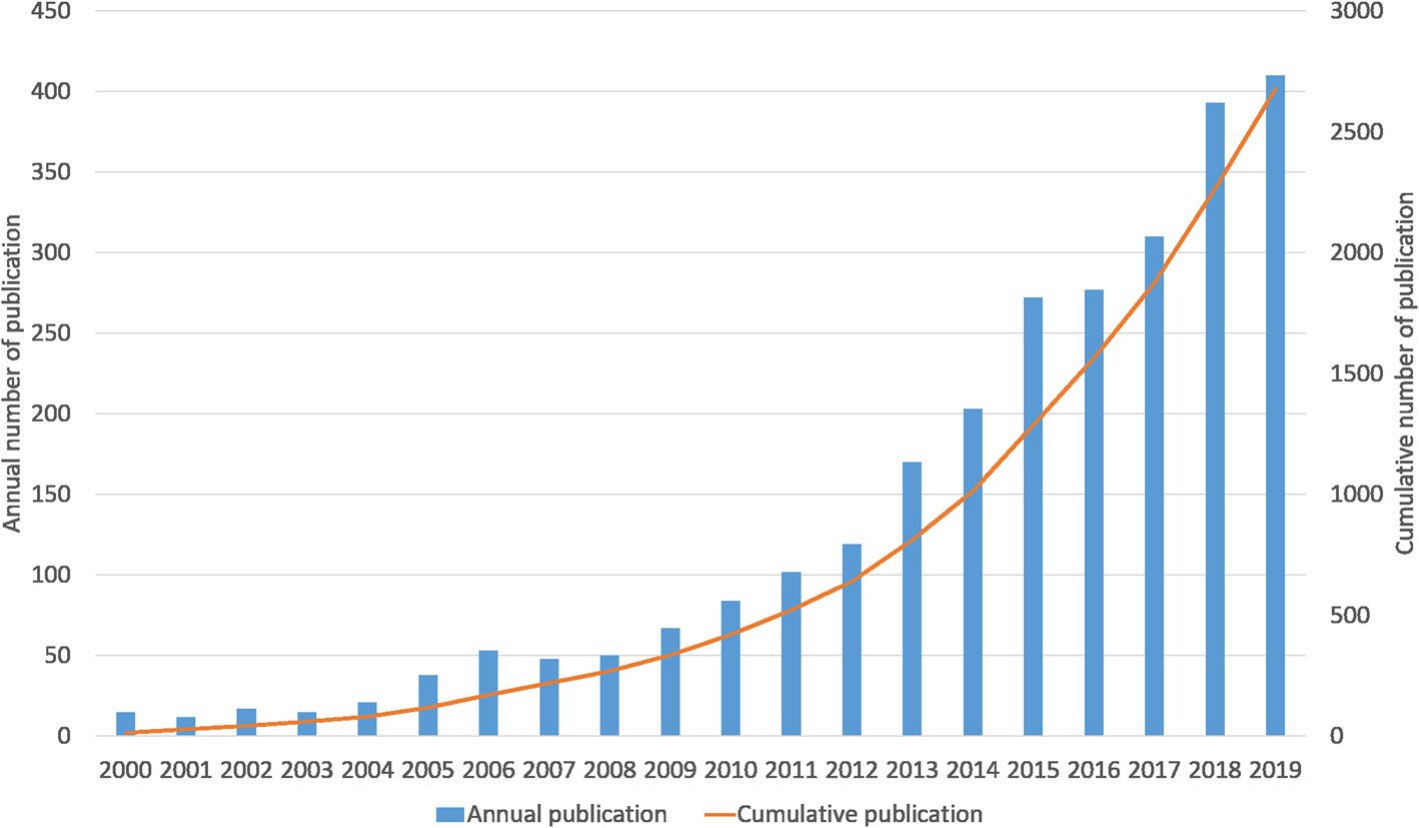
Growth of publication outputs (Horizontal axis: year; Vertical axis: number of publications)

A total of 2,676 publications include 2,442 articles, nine book chapters, 82 proceeding papers, 27 editorial materials, and others. The annual publications increased from 15 in 2000 to 410 in 2019, demonstrating an accelerated rise and upward growth of spatial social network research in the past 20 years. The average annual growth rate of publications in the field was 19.75%. Figure 1 shows that the yearly growth rate of publications has noticeably speeded since 2010.

The average number of authors in a publication increased from 1.933 in 2000 to 3.829 in 2019 (Table 1), which demonstrates that the collaboration has steadily increased. The average number of cited references was generally stable during the 20 years and remained within the range of 47 to 67. Of note, the average citations per article reached its maximum of 191.412 in 2002. Three publications in 2002 had been cited more than 500 times by the end of 2019. Two of these publications were about models for analyzing social networks, specifically latent space approaches (Hoff et al., 2002) and agent-based models (Macy & Willer, 2002), while the third summarized how social network analysis can be applied in information sciences (Otte & Rousseau, 2002).

**Table 1:** Scientific outputs descriptors during 2000–2019

PY	TP	AU	AU/TP	NR	NR/TP	TC	TC/TP
2000	15	29	1.933	980	65.333	1400	93.333
2001	12	24	2.000	834	69.500	1233	102.750
2002	17	33	1.941	981	57.706	3254	191.412
2003	15	32	2.133	714	47.600	1031	68.733
2004	21	39	1.857	1249	59.476	1973	93.952
2005	38	91	2.395	2023	53.237	2957	77.816
2006	53	108	2.038	3539	66.774	7256	136.906
2007	48	109	2.271	2658	55.375	2364	49.250
2008	50	126	2.520	2786	55.720	1948	38.960
2009	67	164	2.448	3404	50.806	3327	49.657
2010	84	207	2.464	4882	58.119	4139	49.274
2011	102	302	2.961	5634	55.235	3929	38.520
2012	119	363	3.050	6321	53.118	4204	35.328
2013	170	577	3.394	9388	55.224	4659	27.406
2014	203	676	3.330	11,629	57.286	4870	23.990
2015	272	943	3.467	15,365	56.489	5026	18.478
2016	277	979	3.534	15,638	56.455	3456	12.477
2017	310	1098	3.542	17,268	55.703	2175	7.016
2018	393	1423	3.621	23,336	59.379	1297	3.300
2019	410	1570	3.829	24,640	60.098	360	0.878

*PY* year, *TP* number of publications, *AU* number of authors, *NR* number of cited references, *TC* total citation count; AU/TP, NR/TP, and *TC/TP* average of authors, references, and citations per paper

### Subject categories and major journals

Spatial social network research has covered a wide variety of themes and many different disciplines. Based on the classification of the Web of Science categories, the most popular categories by order are “Geography” (589 publications, 21.4% of the sample documents), “Environmental Studies” (321 publications, 11.7%), “Computer Science Information Systems” (264 publications, 9.6%), and “Economics” (217 publications, 7.9%). When subject categories are examined, 456 combinations of unique categories were identified. The top 20 combinations of subject categories are illustrated in Table 2. The results show that spatial social network studies are relevant to a wide range of disciplines, while the related research outcomes are mostly rooted in “Geography”, “Multidisciplinary Sciences”, “Geography, Physical; Remote Sensing”, and “Economics; Geography” categories. The most cited publication in the “Geography” group discussed the concept and categories of embeddedness in details (Hess, 2004); in the “Multidisciplinary Sciences” group, a study about the rule for cooperative interactions on social network drew the most citations (Ohtsuki et al., 2006); the most highly cited publication in the “Geography, Physical; Remote Sensing” group used geotagged social media data to monitor visitor use of a national park in Finland and compared the performance with traditional visitor surveys (Heikinheimo et al., 2017); and Giuliani (2007) applied social network analysis to examine the knowledge network structure of wine clusters in Italy and Chile, which received the most citations among publications integrating economics and geography.

**Table 2:** Distribution of the subject category combinations: the top 20

Subject Category Combination	TP(%)	
Geography	235	8.782
Multidisciplinary Sciences	144	5.381
Geography, Physical; Remote Sensing	68	2.541
Economics; Geography	52	1.943
Physics, Multidisciplinary	48	1.794
Green & Sustainable Science & Technology; Environmental Sciences; Environmental Studies	44	1.644
Economics	41	1.532
Sociology	41	1.532
Public, Environmental & Occupational Health	39	1.457
Communication	37	1.383
Environmental Studies; Geography	37	1.383
Computer Science, Information Systems; Geography; Geography, Physical; Information Science & Library Science	35	1.308
Computer Science, Information Systems; Engineering, Electrical & Electronic; Telecommunications	34	1.271
Economics; Environmental Studies; Geography; Regional & Urban Planning	31	1.158
Behavioral Sciences; Zoology	28	1.046
Computer Science, Artificial Intelligence; Computer Science, Information Systems	27	1.009
Computer Science, Interdisciplinary Applications; Engineering, Environmental; Environmental Studies; Geography; Operations Research & Management Science; Regional & Urban Planning	27	1.009
Environmental Studies; Urban Studies	26	0.972
Computer Science, Artificial Intelligence	25	0.934
Ecology	25	0.934
Environmental Studies; Geography; Regional & Urban Planning; Urban Studies	25	0.934
Urban Studies	25	0.934

*TP* number of publications, *%* the percentage of the subject in the study field

The top 20 active journals are summarized in Table 3. In terms of the number of publications, *PLOS ONE* was the most prolific journal, followed by the *International Journal of Geo-Information*, and *Sustainability*. All these three journals are open access journals. Regarding the average citation number per article, the *Journal of Economic Geography*, *Annals of the Association of American Geographers*, and *Urban Studies* were the three most highly cited journals, with magnitudes of 101.800, 40.389, and 39.923, respectively.

**Table 3:** The most active journals

Journals	TP	(%)	TC	(%)	TC/TP
PLOS ONE	77	2.877	1788	2.938	23.221
ISPRS International Journal Of Geo-Information	58	2.167	278	0.457	4.793
Sustainability	44	1.644	123	0.202	2.795
International Journal of Geographical Information Science	35	1.308	442	0.726	12.629
Geoforum	31	1.158	663	1.089	21.387
IEEE Access	29	1.084	59	0.097	2.034
Computers Environment and Urban Systems	27	1.009	449	0.738	16.630
Physica A-Statistical Mechanics and its Applications	27	1.009	348	0.572	12.889
Urban Studies	26	0.972	1038	1.706	39.923
Animal Behaviour	25	0.934	668	1.098	26.720
Scientific Reports	25	0.934	353	0.580	14.120
Applied Geography	22	0.822	440	0.723	20.000
Behavioral Ecology and Sociobiology	21	0.785	450	0.739	21.429
Professional Geographer	21	0.785	301	0.495	14.333
Social Networks	21	0.785	655	1.076	31.190
Transactions in GIS	21	0.785	426	0.700	20.286
Journal of Economic Geography	20	0.747	2036	3.345	101.800
Annals of The Association of American Geographers	18	0.673	727	1.195	40.389
Cities	18	0.673	195	0.320	10.833
European Planning Studies	18	0.673	277	0.455	15.389
Urban Geography	18	0.673	196	0.322	10.889

*TP* number of publications, *TC* total citation count, *TC/TP* average citations per paper

### Geographical and institutional distribution of publications

**Fig. 2:**
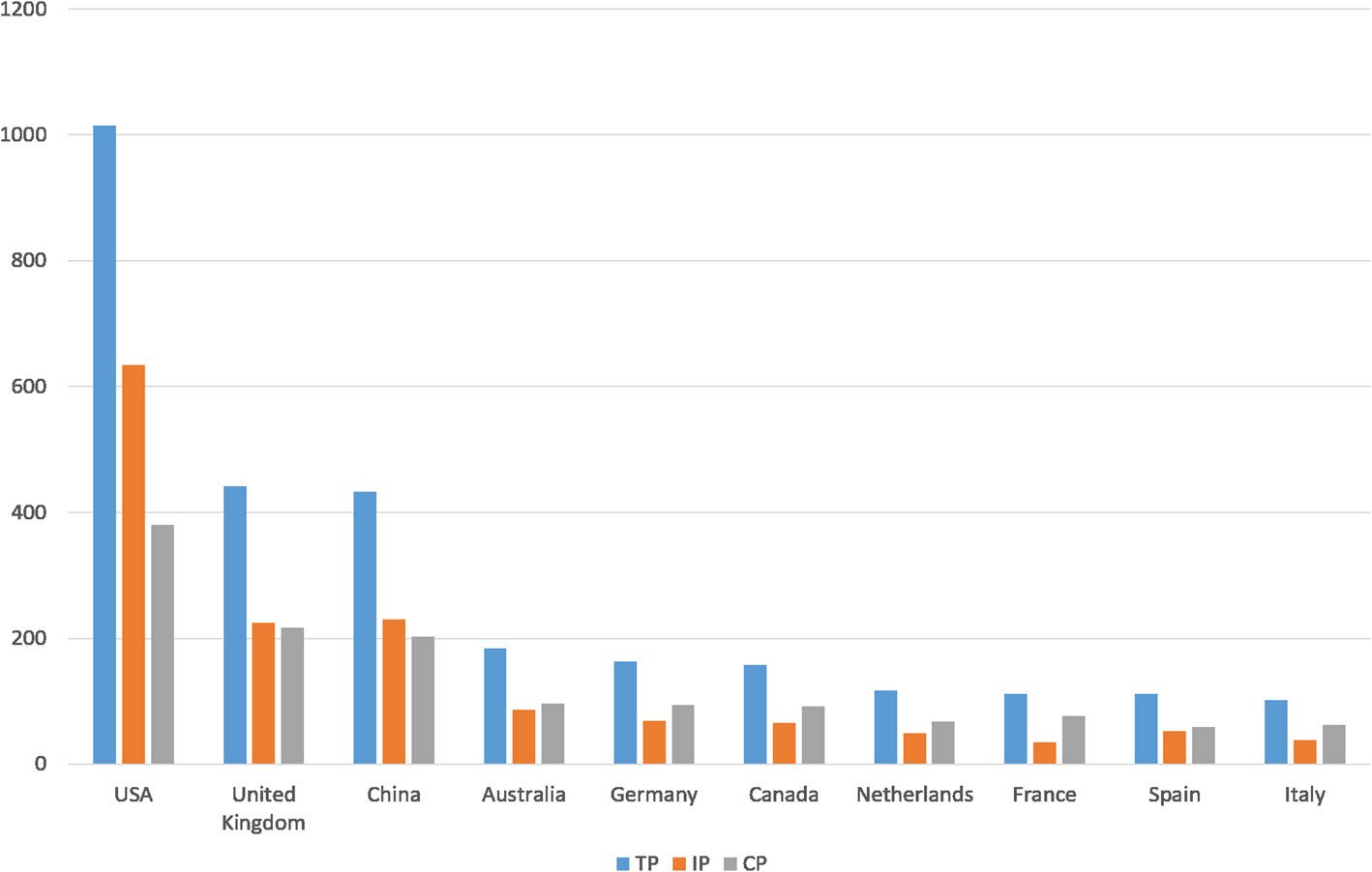
Most productive countries during 2000–2019 (TP: total publications; IP: the number of independent publications by single-country; CP: the number of internationally collaborative publications)

The spatial and institutional distributions of publications were analyzed in terms of authors’ affiliation information. The ten most productive countries are shown in Fig. 2, based on the number of publications, articles by country, and international collaborations. Among these 10 countries, six were located in Europe, two in North America, one in Oceania, and one in Asia.

The most productive country was the United States with 1,015 total articles. The United Kingdom ranked second with 442 articles, followed by China with 433. Figure 2 also reveals that some countries had a higher rate of international collaborations than others. The countries with the highest rates of international collaborations were France (collaboration rate: 68.75%), Italy (collaboration rate: 61.76%), Canada (collaboration rate: 58.21%), the Netherlands (collaboration rate: 58.12%), and Germany (collaboration rate: 57.67%). Almost all were non-native English-speaking countries.

**Fig. 3:**
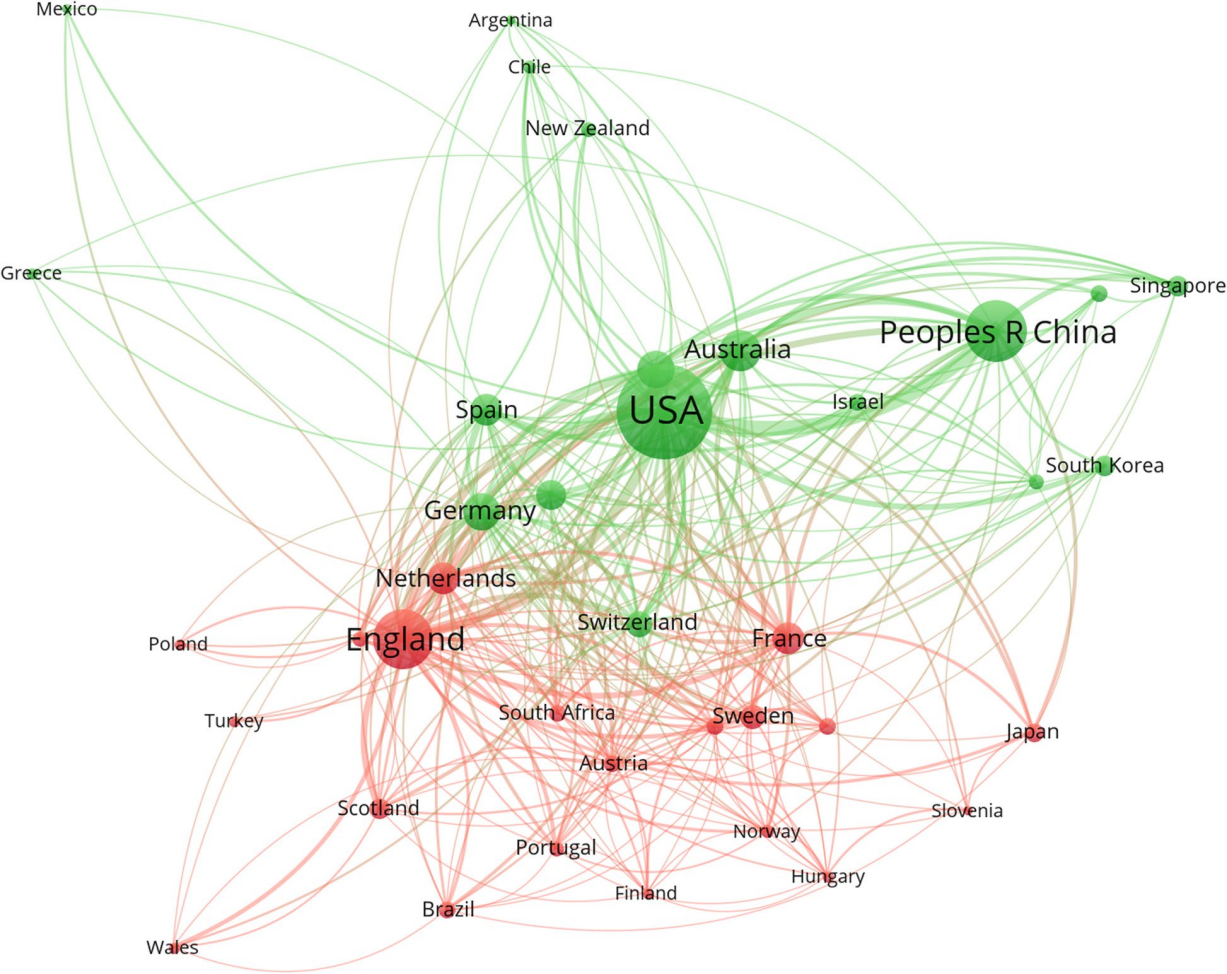
Co-authorship network among productive countries

Co-authorship analysis was used to examine the network of the countries that produced the most research outcomes in the field, as plotted in Fig. 3. The size of the nodes reveals the number of publications with co-authorship in a country, while the thickness of the edges connecting them represents the strength of collaboration. There are two main clusters of collaborations: European countries (the red cluster), and Asian and North American countries (the green cluster). The largest number of papers with co-authorship were yielded by the United States, the United Kingdom, and China. The strongest collaboration was between the United States and China, followed by the United States and England.

### Institution collaboration network

**Fig. 4:**
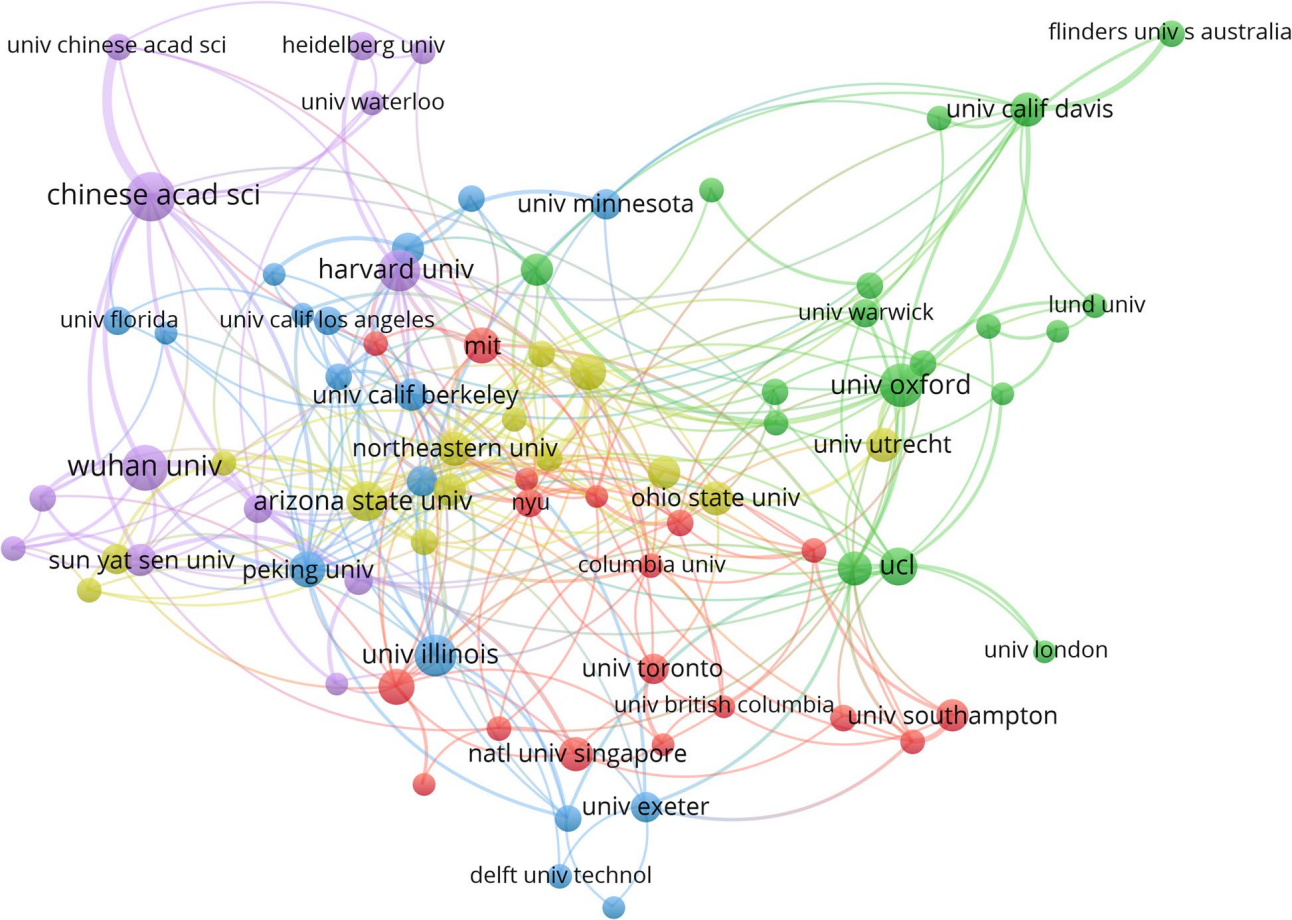
Institution collaboration network of the 80 most productive institutions

The collaboration network of the 80 most productive institutions is visualized based on the VOSviewer (Fig. 4). The most productive institution was Wuhan University with 50 papers, followed by the University of Oxford with 43 papers and Harvard University with 40 papers (see Table 4 for the top 15 most productive institutions). Each node in Fig. 3 indicates an institution of higher education. The distance between two institutions in the visualization roughly represents the relatedness of the institutions in terms of co-authorships. The closer the two institutions are positioned to each other, the stronger their relatedness. The strength of co-authorship links between institutions is also demonstrated by the thickness of edges. The institutions are clustered into five groups of different colors (Fig. 4). Most UK institutions fall in the green group, while the yellow-green groups consist mainly of North American institutions. Most institutions in China fall in the purple group. Institutions within the same continent are more likely to network than institutions from different continents. This means that the geography of institutions maters for collaboration. Nine out of the 10 most highly cited publications that involved institution collaboration were conducted by institutions from the same continent. Researchers from European institutions have co-authored five of these publications (Bastug et al., 2014; Giuliani & Bell, 2005; Gordon & McCann, 2000; Otte & Rousseau, 2002; Perc & Szolnoki, 2010), while collaborations between U.S. institutions have contributed four of these publications (Eagle et al., 2009; Hoff et al., 2002; Macy & Willer, 2002; Sorenson & Stuart, 2001).

**Table 4:** Top 15 institutions based on the total number of publications

Rank	Organization	Country	TP	TC
1	Wuhan University	China	50	278
2	University of Oxford	UK	43	1065
3	Harvard University	US	40	2826
4	Arizona State University	US	39	857
5	University of Illinois	US	38	988
6	Peking University	China	31	630
7	Pennsylvania State University	US	31	515
8	University of Washington	US	31	1505
9	University of Cambridge	UK	29	1292
10	University of California, Davis	US	28	785
11	Northeastern University	US	27	1910
12	Ohio State University	US	27	1037
13	National University of Singapore	Singapore	26	525
14	University of Queensland	Australia	26	556
15	Utrecht University	Netherland	26	1196

TP: number of publications; TC: total citation count

### Keywords analysis

#### Keywords network analysis

**Fig. 5:**
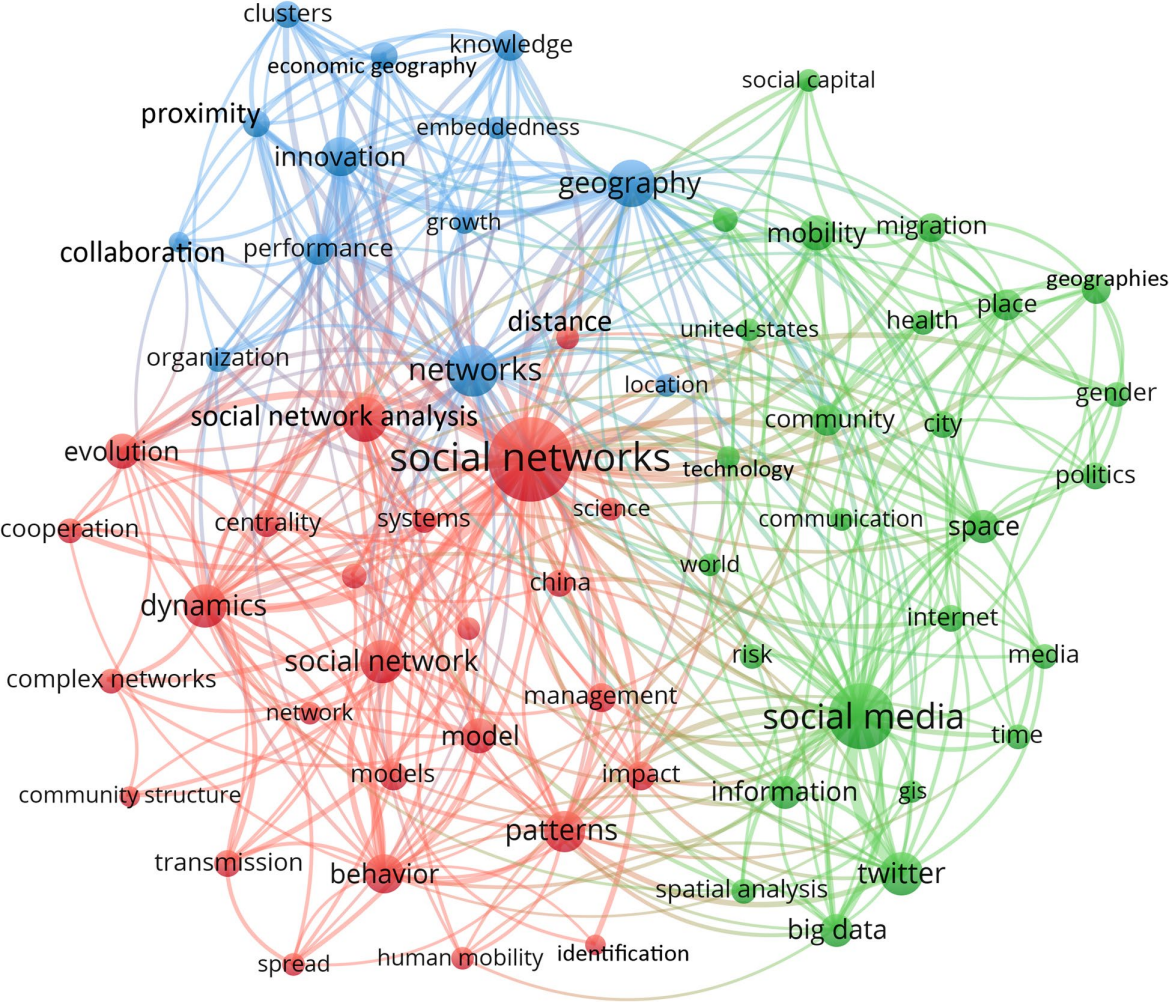
Co-occurrence network of the top 70 high-frequency keywords

Keywords of publications can depict a general profile of the article contents. The co-occurrence relationships among the top 70 high-frequency keywords were explored, and the co-word networks were visualized by the VOSviewer software (Fig. 5). The nodes are high-frequency keywords, whose sizes represent the degree of frequency. The size of the node is larger based on the higher the frequency of keyword use in the last 20 years. The distance between two keywords in the visualization roughly shows the relevance between the keywords regarding the co-occurrence. The closer two keywords are positioned to each other, the stronger their relatedness is. The strength of co-occurrence links between keywords is also demonstrated by the thickness of edges. As shown in Fig. 5, the 70 most frequently used keywords are grouped into three clusters. The red cluster is mainly about social network analysis, the blue cluster is mainly about spatial and geography dimension, and the green cluster is mainly about social media.

The keywords with the highest frequencies were “social networks”, “social media”, “social network analysis”, and “social networks” because they matched the topics we used to collect publications. “Twitter”, “big data”, “networks”, “spatial analysis”, and “social capital” were used more than 50 times by authors, which indicate five research hotspots in the spatial social network field. The most highly cited publications with each of these five keywords were as follows: for “Twitter” research, Takhteyev et al. (2012) investigated different factors for the formation of social ties on Twitter and identified the frequency of airline flights between the two nodes as a vital predictor. Regarding “big data” research, Crampton et al. (2013) analyzed both the potential and shortcomings of big social media data and discussed the impacts of big data analytics on the human geography field. For “networks” research, Macy and Willer (2002) considered that some social network patterns need to be understood using a bottom-up dynamical model, so they introduced agent-based modeling approaches for sociological research. For “spatial analysis” research, a study analyzed the data on shootings in Chicago and Boston, the results of which indicated that both geography and social networks can influence gang violence (Papachristos et al., 2013). For “social capital” research, Carpiano (2006) incorporated the social capital theory into a framework of neighborhood social processes in order to investigate how community factors influence health and well-being. Other popular keywords in the last two decades included “geography”, “network analysis”, “China”, “mobility”, “human mobility”, “migration”, “gender”, “innovation”, “place”, “proximity”, “mobility”, “machine learning”, “GIS”, “Internet”, “location-based social networks”, “algorithms”, “cooperation”, “clustering”, “data mining”, “identity”, “location-based services”, “network”, and “performance”.

#### Temporal evolution of keywords

Examining temporal evolution of these keywords would provide insights about the trends of research hotspots. We divided the 20-year period into three consecutive periods (2000–2009, 2010–2014, and 2015–2019). For the 30 most frequently used keywords mentioned earlier, we listed their frequencies and ranks during the corresponding period in Column (2), (3), and (4) of Table 6.

**Table 6:** Temporal evolution of the 30 most frequently used keywords

DE	(1) Gross		(2) 2000–2009		(3) 2010–2014		(4) 2015–2019		Trend
	N	R	N	R	N	R	N	R	
Social networks	298	1	57	1	104	1	137	2	
Social media	263	2	–-	–-	35	3	228	1	Rising
Social network analysis	166	3	13	3	46	2	107	3	
Social network	112	4	9	6	35	4	68	5	
Twitter	92	5	–-	–-	13	7	79	4	Rising
Big data	63	6	–-	–-	6	22	57	6	Rising
Networks	54	7	17	2	10	14	27	10	
Spatial analysis	52	8	2	104	18	6	32	7	
Social capital	50	9	9	5	19	5	22	14	
Geography	37	10	4	19	10	12	23	11	Rising
Network analysis	36	11	4	22	12	8	20	15	
China	31	12	1	195	3	69	27	8	Rising
Mobility	30	13	5	10	11	10	14	24	
Human mobility	29	14	–-	–-	2	205	27	9	Rising
Migration	27	15	4	21	5	38	18	17	
Gender	25	16	11	4	5	32	9	42	Declining
Innovation	25	17	2	69	8	16	15	22	
Place	25	18	5	12	6	26	14	25	
Proximity	24	19	1	667	11	11	12	32	
Machine learning	23	20	–-	–-	–-	–-	23	12	Rising
GIS	22	21	–-	–-	5	33	17	19	Rising
Internet	22	22	3	30	10	13	9	44	
Location based social networks	22	23	–-	–-	–-	–-	22	13	Rising
Algorithms	20	24	–-	–-	7	19	13	27	
Cooperation	20	25	5	8	11	9	4	157	Declining
Clustering	19	26	1	207	5	29	13	28	Rising
Data mining	19	27	–-	–-	1	678	18	16	Rising
Identity	19	28	5	9	2	206	12	30	
Location based services	19	29	–-	–-	5	37	14	23	Rising
Network	19	30	5	11	8	17	6	102	Declining
Performance	19	31	–-	–-	6	25	13	29	

*DE* keywords, *N* number of articles in the study period, *R* the absolute rank of keywords; –-: no such keyword in the specific time period

If the rank of a keyword keeps moving upward across the three consecutive periods, we consider the keyword to be a rising trend. In contrast, a keyword is in a declining trend if its rank across the three consecutive periods keeps moving downward. It was found that 12 keywords (“social media,” “Twitter,” “big data,” “geography,” “China,” “human mobility,” “machine learning,” “GIS,” “location-based social networks,” “clustering,” “data mining,” and “location-based services”) became increasingly popular in publications during the past 20 years. The keywords “social media,” “Twitter,” and “big data” referred to the data sources of spatial social network analysis. These keywords did not exist in articles in the 2000–2009 period, but they became the first, fourth, and sixth most popular keywords in the period of 2015–2019. This dramatic increase coincided with the popularity of social media and accessibility of social media data. Due to the growing availability of high-speed Internet access and the development of Web 2.0 technology, many social media applications, such as Twitter, Facebook, and YouTube, were created between 2000–2010 (Kaplan & Haenlein, 2010). Twitter provides shorter messaging updates for faster dissemination and application programming interfaces (APIs) for easy data access. As such, Twitter became a valuable and popular tool for researchers to use to collect a large volume of data quickly for little cost (Huberman et al., 2009; Kwak et al., 2010). The spatial component of the spatial social networks is shown in the keywords “geography,” “human mobility,” “GIS,” “location-based social network,” and “location-based service.” Social media often conveyed not only “what” is happening, but also the “where” information, via both user locations and the locations of events (Crooks et al., 2013; Reynard & Shirgaokar, 2019). After some successful demonstrations of the potential of location-based social networks in the early 2010s (Cheng et al., 2011; Cranshaw et al., 2012; Long et al. 2012), many studies started to utilize the spatial component of social network to investigate human mobility patterns. As a result, those “spatial” keywords attracted more attention in the 2010–2014 and 2015–2019 periods. “Machine learning,” “clustering,” and “data mining” represent the popular methods for spatial social network analysis. Social network big data are generated and collected in very high volumes and very quickly and are nearly impossible to be manually read and qualitatively analyzed. Therefore, more and more researchers have started to use data mining (including clustering) and machine learning techniques to discover hidden patterns in such large datasets automatically (Blondel et al., 2015; Jiang et al., 2015; Lansley & Longley, 2016), and even in real time (Gu et al., 2016). The increasing trend of “China” indicates that China has been selected as the study area for spatial social network research more frequently in recent years. In contrast, the keywords “gender”, “cooperation”, and “network” received declining attention across the three time periods. “Gender” and “cooperation” were among the top 10 keywords during 2000–2009. The declining ranks of these keywords could simply reflect the change of research interests in the study field. The change of terminology from general “network” to more specific “social network” or “location-based social network” in later periods could be another possible reason for the declining trend of the keyword “network.”

## Conclusions

Existing studies on research methods have only recently began to pay attention to spatial social network data, although relational data is a longstanding focus in social science. In the past two decades, the annual publications about spatial social network increased from 15 in 2000 to 410 in 2019, with an average annual growth rate of 19.75%. Further, the annual growth rate of publication has greatly accelerated since 2010. The three most productive journals on spatial social network analysis were *PLOS ONE*, *International Journal of Geo-Information*, and *Sustainability*. Regarding average citation number per document, *Journal of Economic Geography*, *Annals of the Association of American Geographers*, and *Urban Studies* were the three most highly cited journals, with average citations per article of 101.800, 40.389, and 39.923, respectively.

The United States was the most productive country, contributing the most single-country and international collaborative articles. The United Kingdom published the second highest number of articles, followed by China. Among the 10 most productive countries, six are in Europe, two are in North America, one is in Oceania, and one is in Asia.

The collaboration network of the top 15 most productive institutes suggest that the Wuhan University, the University of Oxford, and Harvard University were the most productive institutions. Among the 15 institutions, more than half are in the United States. Further, institutions from the same continent collaborate more intensively with one another than with institutions in different continents.

A keywords analysis through temporal evolution and co-occurrence network demonstrated that “Twitter,” “big data,” “networks,” “spatial analysis,” and “social capital” were the long-time keyword hotspots over the past 20 years. Some keywords, such as “social media,” “Twitter,” “big data,” “geography,” “China,” “human mobility,” “machine learning,” “GIS,” “location-based social networks,” “clustering,” “data mining,” and “location-based services,” attracted increasing attention over time.

On the basis of the temporal evolution of keywords, we can conclude that spatial social network analysis research has been enhanced by the rapid development of accessible data sources and big data techniques. Such enhancement facilitated the emerging research directions, such as monitoring human dynamics, building advanced models to solve network-relevant problems, and applying the SNA approach to public health.

1) Monitoring human dynamics. The geotagged user-generated information obtained from social media platforms (e.g., Twitter, Facebook, and Instagram) greatly facilitate human mobility and migration monitoring (Hu et al., 2021a). Facebook developed the Data for Good Movement Range Maps, to notify scholars and public health practitioners how people act upon physical distancing measures (Meta, 2022). Huang et al. (2020) created a mobility-based responsive index derived from geotagged Twitter data to monitor human mobility. The team further developed an online platform, share the mobility index with the public (Li et al., 2021). The accessible human mobility datasets dramatically promoted the emergence preparedness and responses. For example, Bonaccorsi et al. (2020) performed a massive analysis on near-real-time mobility data provided by Facebook and investigated how lockdown strategies impact the development of economics. Huang et al. (2021) analyzed multi-source human mobility data and highlighted the disparities in mobility dynamics across counties of various income levels in the US during the COVID-19 pandemic.

2) Advanced modeling in spatial social networks. With the rapid advances of Artificial Intelligence (AI), many studies utilize deep learning approaches to solve graph-related problems, such as GraphSage (Ahmed et al., 2017), GAAN (Zhang et al., 2018), and DeepMGGE (Fu et al., 2020). In social network, spatial variation is a key factor as well as the temporal-evolution characteristics. Thus, recent studies considered both spatial and temporal features to model the social networks. For instance, Min et al. (2021) utilized the temporal attention mechanism to identify the dynamic features of social networks and also proposed Spatial–Temporal Graph Social Network, a graph neural network framework. The method outperformed state-of-the-art machine learning algorithms.

3) Applications in public health. SSNA has been widely applied in subjects of geography (Wang et al, 2022a, b), economics (Bu et al., 2020; Reid et al., 2008), computer science (Fu et al., 2020; Min et al., 2021), information science (Hu & Zhang, 2021; Ye & Andris, 2021), and urban planning (Ye & Liu, 2018). The recent COVID-19 pandemic accelerates the application of SSNA in public health as well, especially in information distribution (Ye et al, 2021), public sentiments and opinions (Gong & Ye, 2021; Hu et al., 2021b; Yang et al., 2022), and disease modeling (Yum, 2020; Albery et al. 2021). For example, Ye et al. (2021) incorporated information heterogeneity into non-parametric inference of the hidden interaction network to understand both infodemic and epidemic spreading in the COVID-19 pandemic (Ye et al., 2021). Block et al. (2020) adopted s social network approach to assess the effectiveness of social distancing strategies in the COVID-19 pandemic.

## Data Availability

Not applicable.

## References

[CR1] Ahmed, N. K., Rossi, R. A., Zhou, R., Lee, J. B., Kong, X., Willke, T. L., & Eldardiry, H. (2017). Inductive representation learning in large attributed graphs. arXiv preprint arXiv:1710.09471.

[CR2] Albery GF, Kirkpatrick L, Firth JA, Bansal S (2021). Unifying spatial and social network analysis in disease ecology. Journal of Animal Ecology.

[CR3] Aria M, Cuccurullo C (2017). bibliometrix: An R-tool for comprehensive science mapping analysis. Journal of Informetrics.

[CR4] Bastug E, Bennis M, Debbah M (2014). Living on the edge: The role of proactive caching in 5G wireless networks. IEEE Communications Magazine.

[CR5] Block P, Hoffman M, Raabe IJ, Dowd JB, Rahal C, Kashyap R, Mills MC (2020). Social network-based distancing strategies to flatten the COVID-19 curve in a post-lockdown world. Nature Human Behaviour.

[CR6] Blondel VD, Decuyper A, Krings G (2015). A survey of results on mobile phone datasets analysis. EPJ Data Science.

[CR7] Bonaccorsi G, Pierri F, Cinelli M, Flori A, Galeazzi A, Porcelli F, Pammolli F (2020). Economic and social consequences of human mobility restrictions under COVID-19. Proceedings of the National Academy of Sciences.

[CR8] Bu Y, Wang E, Bai J, Shi Q (2020). Spatial pattern and driving factors for interprovincial natural gas consumption in China: Based on SNA and LMDI. J Cleaner Production.

[CR9] Carpiano RM (2006). Toward a neighborhood resource-based theory of social capital for health: Can Bourdieu and sociology help?. Social Science and Medicine.

[CR10] Cheng Z, Caverlee J, Lee K, Sui DZ, Nicolov N, Shanahan JG (2011). Exploring millions of footprints in location sharing services. Proceedings of the Fifth International AAAI Conference on Weblogs and Social Media.

[CR11] Crampton JW, Graham M, Poorthuis A, Shelton T, Stephens M, Wilson MW, Zook M (2013). Beyond the geotag: Situating “big data” and leveraging the potential of the geoweb. Cartography and Geographic Information Science.

[CR12] Cranshaw J, Schwartz R, Hong J, Sadeh N, Breslin JG, Ellison NB, Shanahan JG, Tufekci Z (2012). The Livehoods Project: Utilizing social media to understand the dynamics of a city. Proceedings of the Sixth International AAAI Conference on Weblogs and Social Media.

[CR13] Crooks A, Croitoru A, Stefanidis A, Radzikowski J (2013). #Earthquake: Twitter as a distributed sensor system. Transactions in GIS.

[CR14] Ding Y, Chowdhury GG, Foo S (2001). Bibliometric cartography of information retrieval research by using co-word analysis. Information processing & management.

[CR15] Donthu N, Kumar S, Mukherjee D, Pandey N, Lim WM (2021). How to conduct a bibliometric analysis: An overview and guidelines. Journal of Business Research.

[CR16] Eagle N, Pentland A, Lazer D (2009). Inferring friendship network structure by using mobile phone data. Proceedings of the National Academy of Sciences of the United States of America.

[CR17] Emch M, Root ED, Giebultowicz S, Ali M, Perez-Heydrich C, Yunus M (2012). Integration of spatial and social network analysis in disease transmission studies. Annals of the Association of American Geographers.

[CR18] Fu S, Wang G, Xia S, Liu L (2020). Deep multi-granularity graph embedding for user identity linkage across social networks. Knowledge-Based Systems.

[CR19] Garfield E (1970). Citation indexing for studying science. Nature.

[CR20] Giuliani E (2007). The selective nature of knowledge networks in clusters: Evidence from the wine industry. Journal of Economic Geography.

[CR21] Giuliani E, Bell M (2005). The micro-determinants of meso-level learning and innovation: Evidence from a Chilean wine cluster. Research Policy.

[CR22] Glänzel, W., & Schubert, A. (2004). *Analysing scientific networks through co-authorship*. Springer.

[CR23] Gong X, Ye X (2021). Governors Fighting Crisis: Responses to the COVID-19 Pandemic across US States on Twitter. The Professional Geographer.

[CR24] Gordon IR, McCann P (2000). Industrial Clusters: Complexes, Agglomeration and/or Social Networks?. Urban Studies.

[CR25] Gu Y, Qian Z, Chen F (2016). From Twitter to detector: Real-time traffic incident detection using social media data. Transportation Research Part c: Emerging Technologies.

[CR26] He Y, Hui SC (2002). Mining a web citation database for author co-citation analysis. Information Processing & Management.

[CR27] Heikinheimo V, Di Minin E, Tenkanen H, Hausmann A, Erkkonen J, Toivonen T (2017). User-generated geographic information for visitor monitoring in a national park: A comparison of social media data and visitor survey. ISPRS International Journal of Geo-Information.

[CR28] Hess M (2004). ‘Spatial’ relationships? Towards a reconceptualization of embeddedness. Progress in Human Geography.

[CR29] Hoff PD, Raftery AE, Handcock MS (2002). Latent space approaches to social network analysis. Journal of the American Statistical Association.

[CR30] Huang, X., Li, Z., Jiang, Y., Li, X., & Porter, D. (2020). Twitter reveals human mobility dynamics during the COVID-19 pandemic. *PloS one, 15*(11), e0241957. 10.1371/journal.pone.0241957 PMC7654838 33170889

[CR31] Huang, X., Li, Z., Jiang, Y., Ye, X., Deng, C., Zhang, J., & Li, X. (2021). The characteristics of multi-source mobility datasets and how they reveal the luxury nature of social distancing in the US during the COVID-19 pandemic. *International Journal of Digital Earth, 14*(4), 424–442.

[CR32] Hu T, Zhang Y (2021). A spatial–temporal network analysis of patent transfers from US universities to firms. Scientometrics.

[CR33] Hu T, Wang S, Luo W, Zhang M, Huang X, Yan Y, Li Z (2021). Revealing Public Opinion Towards COVID-19 Vaccines with Twitter Data in the United States: Spatiotemporal Perspective. Journal of Medical Internet Research.

[CR34] Hu T, Wang S, She B, Zhang M, Huang X, Cui Y, Li Z (2021). Human mobility data in the COVID-19 pandemic: Characteristics, applications, and challenges. International Journal of Digital Earth.

[CR35] Huberman BA, Romero DM, Wu F (2009). Social networks that matter Twitter under the microscope. First Monday.

[CR36] Jiang S, Alves A, Rodrigues F, Ferreira J, Pereira FC (2015). Mining point-of-interest data from social networks for urban land use classification and disaggregation. Computers, Environment and Urban Systems.

[CR37] Kaplan AM, Haenlein M (2010). Users of the world, unite! The challenges and opportunities of Social Media. Business Horizons.

[CR38] Kwak H, Lee C, Park H, Moon S, Rappa M, Jones P, Freire J, Chakrabarti S (2010). What is Twitter, a social network or a news media?. Proceedings of the 19th International Conference on World Wide Web, WWW ’10.

[CR39] Lansley G, Longley PA (2016). The geography of Twitter topics in London. Computers, Environment and Urban Systems.

[CR40] Li Q, Wei W, Xiong N, Feng D, Ye X, Jiang Y (2017). Social media research, human behavior, and sustainable society. Sustainability.

[CR41] Li Z, Huang X, Hu T, Ning H, Ye X, Huang B, Li X (2021). ODT FLOW: Extracting, analyzing, and sharing multi-source multi-scale human mobility. PLoS ONE.

[CR42] Long X, Jin L, Joshi J., Dey AK, Chu H, Hayes G (2012). Exploring trajectory-driven local geographic topics in foursquare. Proceedings of the 2012 ACM Conference on Ubiquitous Computing, UbiComp’12.

[CR43] Macy MW, Willer R (2002). From Factors to Factors: Computational Sociology and Agent-Based Modeling. Annual Review of Sociology.

[CR44] Meta, 2022. https://dataforgood.facebook.com/dfg/tools/movement-range-maps. Accessed 2 May 2022

[CR45] Min S, Gao Z, Peng J, Wang L, Qin K, Fang B (2021). STGSN—A Spatial-Temporal Graph Neural Network framework for time-evolving social networks. Knowledge-Based Systems.

[CR46] Ohtsuki H, Hauert C, Lieberman E, Nowak MA (2006). A simple rule for the evolution of cooperation on graphs and social networks. Nature.

[CR47] Otte E, Rousseau R (2002). Social network analysis: A powerful strategy, also for the information sciences. Journal of Information Science.

[CR48] Papachristos AV, Hureau DM, Braga AA (2013). The Corner and the Crew: The Influence of Geography and Social Networks on Gang Violence. American Sociological Review.

[CR49] Perc M, Szolnoki A (2010). Coevolutionary games—A mini review. Bio Systems.

[CR50] Pritchard A (1969). Statistical bibliography or bibliometrics. Journal of documentation.

[CR51] Reid N, Smith BW, Carroll MC (2008). Cluster regions: A social network perspective. Economic Development Quarterly.

[CR52] Reynard D, Shirgaokar M (2019). Harnessing the power of machine learning: Can Twitter data be useful in guiding resource allocation decisions during a natural disaster?. Transportation Research Part D: Transport and Environment.

[CR53] Schmoch U, Schubert T (2008). Are international co-publications an indicator for quality of scientific research?. Scientometrics.

[CR54] Scott J (1988). Social network analysis. Sociology.

[CR55] Scott J (2000). Social network analysis: A handbook. Contemporary Sociology.

[CR56] Song J, Feng Q, Wang X, Fu H, Jiang W, Chen B (2018). Spatial association and effect evaluation of CO2 emission in the Chengdu-Chongqing urban agglomeration: Quantitative evidence from social network analysis. Sustainability.

[CR57] Sorenson O, Stuart TE (2001). Syndication networks and the spatial distribution of venture capital investments. American Journal of Sociology.

[CR58] Sun Q, Wang N, Li S, Zhou H (2018). Local spatial obesity analysis and estimation using online social network sensors. Journal of Biomedical Informatics.

[CR59] Takhteyev Y, Gruzd A, Wellman B (2012). Geography of Twitter networks. Social Networks.

[CR60] Tsou MH, Yang JA, Richardson D (2016). Spatial social networks. The international encyclopedia of geography.

[CR61] Van Eck N, Waltman L (2009). Software survey: VOSviewer, a computer program for bibliometric mapping. Scientometrics.

[CR62] Wang, P., Hu, T., Gao, F., Wu, R., Guo, W., & Zhu, X. (2022a). A Hybrid Data-Driven Framework for Spatiotemporal Traffic Flow Data Imputation. *IEEE Internet Things Journal*. 10.1109/JIOT.2022.3151238.

[CR63] Wang, P., Zhang, T., Zheng, Y., & Hu, T. (2022b). A multi-view bidirectional spatiotemporal graph network for urban traffic flow imputation. *International Journal of Geographical Information Science*, *36*(6), 1231–1257.

[CR64] Yang X, Ye X, Sui DZ (2016). We know where you are: In space and place-enriching the geographical context through social media. International Journal of Applied Geospatial Research.

[CR65] Yang YX, Zhang YY, Zhang XW, Cao YH, Zhang J (2022). Spatial evolution patterns of public panic on Chinese social networks amidst the COVID-19 pandemic. International Journal of Disaster Risk Reduction.

[CR66] Ye X, Andris C (2021). Spatial social networks in geographic information science. International Journal of Geographical Information Science.

[CR67] Ye X, Liu X (2018). Integrating social networks and spatial analyses of the built environment. Environment and Planning B: Urban Analytics and City Science.

[CR68] Ye X, Wang W, Zhang X, Li Z, Yu D, Du J, Chen Z (2021). Reconstructing spatial information diffusion networks with heterogeneous agents and text contents. Transactions in GIS.

[CR69] Yum S (2020). Social network analysis for coronavirus (COVID-19) in the United States. Social Science Quarterly.

[CR70] Zhang P, Zhao Y, Zhu X, Cai Z, Xu J, Shi S (2020). Spatial structure of urban agglomeration under the impact of high-speed railway construction: Based on the social network analysis. Sustainable Cities Soc.

[CR71] Zhang, J., Shi, X., Xie, J., Ma, H., King, I., & Yeung, D. Y. (2018). Gaan: Gated attention networks for learning on large and spatiotemporal graphs. arXiv preprint arXiv:1803.07294.

[CR72] Zhu X, Wang Q, Zhang P, Yu Y, Xie L (2021). Optimizing the spatial structure of urban agglomeration: Based on social network analysis. Quality & Quantity.

